# Predictive accuracy of elevated mitotic rate on lymph node positivity and recurrence in thin melanomas

**DOI:** 10.3389/fonc.2022.1077226

**Published:** 2023-01-06

**Authors:** Catherine L. Ly, Ondrej Blaha, Wei Wei, Anjela Galan, Harriet Kluger, Stephan Ariyan, Kelly Olino, James E. Clune

**Affiliations:** ^1^ Division of Plastic and Reconstructive Surgery, Department of Surgery, Yale School of Medicine, New Haven, CT, United States; ^2^ Department of Biostatistics, Yale School of Public Health, New Haven, CT, United States; ^3^ Departments of Dermatology and Pathology, Yale School of Medicine, New Haven, CT, United States; ^4^ Division of Medical Oncology, Department of Internal Medicine, Yale School of Medicine, New Haven, CT, United States; ^5^ Division of Surgical Oncology, Department of Surgery, Yale School of Medicine, New Haven, CT, United States

**Keywords:** melanoma, sentinel lymph node biopsy (SLNB), recurrence, mitotic rate, skin cancer

## Abstract

**Background:**

Mitotic rate (MR) is considered an important prognostic factor for melanoma but is not currently used for staging because its nuanced effect is not yet well-delineated. We sought to determine if T category-specific MR is predictive of sentinel lymph node (SLN) positivity, recurrence, and melanoma-specific mortality (MSM).

**Methods:**

A retrospective review of patients with primary cutaneous melanoma from 1994 to 2020 at a single academic center was performed. Patient demographics and tumor characteristics were recorded. MR was considered elevated for each AJCC8-defined T category if it was ≥2 mitoses/mm^2^ for T1, ≥4 mitoses/mm^2^ for T2, ≥6 mitoses/mm^2^ for T3, or ≥7 mitoses/mm^2^ for T4. Statistical analysis was performed to assess the predictive accuracy of MR on selected outcomes while controlling for ulceration.

**Results:**

Data from 2,984 patients with complete records were analyzed. Along with Breslow thickness and ulceration, elevated MR was associated with higher risk of MSM (HR 1.816, *P*=0.0001). There was no difference among patients with ulcerated T1 or T2 tumors regardless of MR, but those with non-ulcerated T1 or T2 tumors and elevated MR were more likely to have positive SLNs (P<0.0001 and *P*=0.0043, respectively) and recurrence (*P*=0.0007 and *P*=0.0004, respectively) compared to counterparts with low MR. There were no notable differences for T3 or T4 tumors based on MR.

**Conclusions:**

Elevated MR is associated with SLN positivity and recurrence in thin melanomas, independent of ulceration. SLN biopsy should therefore be strongly considered for patients with non-ulcerated lesions <0.8 mm thick if the MR is ≥2 mitoses/mm^2^.

## Introduction

A clear understanding of the prognostic factors for malignant cutaneous melanoma is becoming increasingly paramount as its incidence continues to rise in the United States (1). Strong evidence has demonstrated that greater Breslow thickness and ulceration are correlated with melanoma-specific mortality (MSM), resulting in their inclusion in the staging system. Mitotic rate (MR) also contributes to melanoma outcomes, but its exact role remains poorly elucidated (2–7).

The seventh edition of the American Joint Committee on Cancer (AJCC) staging system for primary cutaneous melanoma (AJCC7) incorporated MR as a high-risk feature for T1 melanomas (≤1 mm thick); tumors were upstaged from T1a to T1b should they demonstrate ulceration or MR ≥1 mitosis/mm^2^ (8). However, MR was removed from the staging system in the subsequent eighth edition (AJCC8), in which tumors are characterized as T1b if they are either 0.8-1.0 mm thick without ulceration or ≤1 mm with ulceration (6, 9). This was determined after a multivariate analysis of 7,568 patients with T1 melanoma without nodal metastasis found that thickness ≥0.8 mm and ulceration were more powerful predictors of melanoma-specific mortality (MSM) than MR when it is treated as a dichotomous variable (<1 or ≥1 mitosis/mm^2^). There were additional concerns that the AJCC7 system had caused pathologists to look more carefully for a single mitotic factor, potentially resulting in morbidity in patients who may not have necessarily otherwise been upstaged (10). Despite these findings, additional analyses demonstrated that MR is a significant predictor when utilized across its dynamic range and that increased MR is indeed likely associated with an increased risk of sentinel lymph node (SLN) metastasis. The Melanoma Expert Panel therefore recommended that MR should continue to be collected in order to aid research aimed at identifying the best means by which to characterize its effect on melanoma outcomes (7).

The importance of MR is supported in a body of literature published since AJCC8, but each of these studies has utilized differing cut-points for MR to evaluate its role (2, 4, 5, 11, 12). One of the largest and most robust studies was performed by Kashani-Sabet et al., who evaluated 5,050 patients from two populations who had either died from metastatic melanoma any time after their initial diagnosis or had at least eight years of follow-up without evidence of distant metastasis (11). These authors constructed the following computer-generated cut-points for MR for each T stage to determine its impact on MSM: <2 mitoses/mm^2^ and ≥2 mitoses/mm^2^ for T1; <4 mitoses/mm^2^ and ≥4 mitoses/mm^2^ for T2; <6 mitoses/mm^2^ and ≥6 mitoses/mm^2^ for T3; and <7 mitoses/mm^2^ and ≥7 mitoses/mm^2^ for T4. They concluded that there is a nonlinear relationship between MR and MSM and that the subsequent AJCC staging system should re-incorporate MR. In our study, we sought to not only validate the use of MR using the cut-points determined by Kashani-Sabel et al., but also to determine the effect of MR on SLN positivity and recurrence.

## Materials and methods

### Patient selection

Adult patients diagnosed with and treated for malignant cutaneous melanoma were identified through a retrospective review of patients seen at the Yale-New Haven Hospital Smilow Cancer Center between January 1994 and August 2020 (IRB #2000029420). All patient data are tracked in a prospectively maintained melanoma tumor registry at this high-volume institution. Patients were included if they were over 18 years of age, had a histologically confirmed diagnosis of a single cutaneous melanoma with complete data on tumor characteristics (Breslow thickness, ulceration, and MR), and at least one documented follow-up. Patients were excluded if they had multiple primary cutaneous melanoma sites, uveal or mucosal melanoma, or missing information on tumor characteristics.

### Data variables and primary outcomes

Patient charts were examined for age at initial diagnosis, gender, living status, Breslow thickness, ulceration, MR, number of positive lymph nodes if the patient underwent SLN biopsy (SLNB), site of recurrence if applicable, cause of death if applicable, and time of latest follow-up. The decision to perform SLNB for each patient was made following individualized discussions of the risks and benefits with the guidance of both the recommendations put forth by the American Society of Clinical Oncology (ASCO) and Society of Surgical Oncology (SSO) at the time of diagnosis and the multidisciplinary team’s clinical expertise (13). For the purposes of this study, all tumors were re-categorized based on the most recent guidelines (AJCC8).

MR was determined by experienced pathologists using a standard protocol (14, 15). It is reported per 1 mm^2^ and is performed by finding the hot spot field in the invasive melanoma component and by counting the mitoses within it and adjacent non-overlapping fields to achieve a surface of 1 mm. If the tumor is large, the mitoses are counted in several 1 mm fields, then the total number of mitoses is added then divided by the number of fields to obtain the average mitotic count. If the tumor is small (<1 mm), the number of found mitoses is reported per 1 mm^2^. MR was then categorized based on T-specific cut-points put forward by Kashani-Sabet et al. and was considered elevated if it was ≥2 mitoses/mm^2^ for T1, ≥4 mitoses/mm^2^ for T2, ≥6 mitoses/mm^2^ for T3, or ≥7 mitoses/mm^2^ for T4 (11).

### Statistical analysis

All statistical analyses were performed using R software (Version 4.1.2; Foundation for Statistical Computing; Vienna, Austria). A p-value (*P*) of less than 0.05 was considered statistically significant. All descriptive statistics are reported as mean (sd) for continuous and as frequency (%) for categorical variables. Multivariate Cox regression analysis was adjusted for Breslow thickness and ulceration, the two factors that define T category in the AJCC8. Age and gender were also taken into consideration as evidence has shown worse outcomes with increased age at diagnosis and male gender (16, 17). The time-to-event data were analyzed using multivariate proportional hazards model and the survival plots were generated using the Kaplan-Meier estimation method. The association in the frequency tables was tested *via* z-test or Fisher’s exact test wherever appropriate.

## Results

A total of 3,052 patients with histologically confirmed primary cutaneous melanoma were identified in the melanoma tumor registry. After patients with incomplete records were excluded, 2,984 patients remained for analysis. The patients’ demographic and clinical characteristics are presented in **Table 1**. Slightly more patients were male (55.1%) and the majority was between the ages of 41 and 80 years (77.2%) at initial diagnosis. An overwhelming majority was alive at last follow-up (81.2%) and of those who were deceased, 37.6% had died specifically of melanoma. The mean length of follow-up was 5.3 years (range 0-24 years), which is consistent with our standard-of-care protocol for 5-year follow-up for all patients diagnosed with melanoma. Histologic analysis demonstrated that more than half of the patients had T1 tumors and the majority of tumors were not ulcerated (81.8%) or with an elevated MR (79.5%). The majority were confirmed to be superficial spreading (48.2%) or nodular melanoma (10.7%); very few had acral lentiginous melanoma (0.13%) in this cohort.

**Table 1:** Patient demographics and clinical characteristics.

	n (%)
Age at diagnosis (years)	
≤40	306 (10.3)
41-60	1035 (34.7)
61-80	1268 (42.5)
>80	375 (12.6)
Gender	
Male	1643 (55.1)
Female	1341 (44.9)
Clinical status	
Alive	2431 (81.5)
Dead	553 (18.5)
Cause of death	
Melanoma	208 (7.0)
Co-morbidities	153 (5.1)
Other cancer	40 (1.3)
Unknown	152 (5.1)
Breslow thickness (mm)	
≤1.0	1580 (52.9)
1.1-2.0	644 (21.6)
2.1-4.0	401 (13.4)
>4.0	359 (12.0)
Ulceration	
No	2440 (81.8)
Yes	544 (18.2)
Elevated mitotic rate	
No	2373 (79.5)
Yes	611 (20.5)

We first performed multivariate Cox regression analysis to validate Kashani-Sabet et al.’s conclusion that MR is an independent predictive factor for MSM. Consistent with their findings, we found that MR evaluated either using the entire scale or as a binary variable utilizing the aforementioned cut-points was independently predictive of survival (HR 1.022, *P*=0.0064 and HR 1.816, *P*=0.0001, respectively) (**Tables 2**, **3**). As with Kashani-Sabet et al.’s results, the likelihood ratio chi-square statistic was higher when MR was analyzed as a binary factor, indicating improved fitness compared to when MR was used as a continuous factor. Age, gender, Breslow thickness, and ulceration were also significant in both analyses as expected.

**Table 2:** Effect of mitotic rate as a continuous variable on melanoma-specific mortality.

Covariate	Chi-Square Statistic	Hazard Ratio	*P*
**Age**	9.524	1.015	0.0025
**Male gender**	9.971	1.613	0.0021
**Thickness**	48.622	1.106	<0.0001
**Ulceration**	63.860	3.502	<0.0001
**Mitotic rate**	6.317	1.022	0.0064

Likelihood ratio chi-square, 256.2; *P*<0.0001

**Table 3:** Effect of mitotic rate as a binary variable on melanoma-specific mortality.

Covariate	Chi-Square Statistic	Hazard Ratio	*P*
**Age**	11.978	1.017	0.0007
**Male gender**	10.743	1.640	0.0014
**Thickness**	61.843	1.112	<0.0001
**Ulceration**	52.613	3.167	<0.0001
**Mitotic rate**	14.654	1.816	0.0001

Likelihood ratio chi-square, 264.6; *P*<0.0001

We then sought to determine the impact of elevated MR on SLN positivity for each T category (**Table 4**). To eliminate the potential confounding effect of ulceration, low and high MR groups were further divided into non-ulcerated and ulcerated tumors. Among T1 tumors, there was no difference among patients with ulcerated tumors regardless of MR (*P*=0.17) but non-ulcerated tumors with elevated MR were more likely to have positive SLNs compared to those with low MR (*P*<0.0001). Slightly more than half of these patients with non-ulcerated tumors and elevated MR (n=85, 51.2%) underwent SLNB with a 14.1% positivity rate. Of note, 15 of these patients had a Breslow thickness <0.8 mm and, of these, 20% had positive SLNs (data not shown). Patients with T2 tumors had similar findings; those with ulcerated tumors were not different based on MR (*P*>0.9999), whereas those with non-ulcerated tumors and elevated MR were more likely to have positive SLNs compared to their low MR counterparts (*P*=0.0043). Interestingly, analyses for T3 and T4 tumors did not demonstrate any significant differences between low and high MR.

**Table 4:** Sentinel lymph node positivity, recurrence, and melanoma-specific mortality among patients separated by mitotic rate and ulceration.

MR	Ulceration	n (%)	+SLNB (% total SLNB)	Recurrence (%)	MSM (%)
T1 (n=1580)					
<2	No	1374 (87.0)	15 (7.4)	33 (2.4)	12 (0.9)
	Yes	23 (1.5)	0 (0)	4 (17.4)	0 (0.0)
≥2	No	166 (10.5)	12 (14.1)	13 (7.8)	6 (3.6)
	Yes	17 (1.1)	2 (16.7)	1 (5.9)	0 (0.0)
*P*	No		<0.0001	0.0007	0.0089
	Yes		0.1744	0.3725	1.0000
T2 (n=644)					
<4	No	437 (67.9)	37 (9.7)	51 (11.7)	20 (4.58)
	Yes	76 (11.8)	12 (17.9)	10 (13.2)	3 (3.95)
≥4	No	95 (14.75)	18 (23.7)	25 (26.3)	7 (7.4)
	Yes	36 (5.6)	6 (21.4)	8 (22.2)	4 (11.1)
*P*	No		0.0043	0.0004	0.2990
	Yes		>0.9999	0.3450	0.2086
T3 (n=401)					
<6	No	177 (44.1)	43 (28.5)	46 (26.0)	17 (9.6)
	Yes	109 (27.2)	25 (29.1)	49 (45.0)	22 (20.18)
≥6	No	55 (13.72)	14 (30.43)	18 (32.7)	9 (16.36)
	Yes	60 (15.0)	16 (34.8)	29 (48.3)	14 (23.3)
*P*	No		>0.9999	0.4214	0.2592
	Yes		0.7234	0.7945	0.7777
T4 (n=359)					
<7	No	90 (25.1)	25 (36.8)	36 (40.0)	22 (24.4)
	Yes	87 (24.2)	23 (38.3)	46 (52.9)	23 (26.4)
≥7	No	46 (12.8)	19 (55.9)	22 (47.8)	9 (19.6)
	Yes	136 (37.9)	34 (42.0)	73 (53.7)	32 (23.5)
*P*	No		0.1610	0.4903	0.6703
	Yes		0.9342	>0.9999	0.7398

MR, mitotic rate; MSM, melanoma-specific mortality; SLNB, sentinel lymph node biopsy

A corresponding analysis was performed for recurrence and yielded similar results in which those with non-ulcerated T1 and T2 tumors with elevated MR were more likely to have recurrence compared to counterparts with low MR (*P*=0.0007 and *P*=0.0004, respectively) (**Table 4**). Looking more carefully, non-ulcerated T1 tumors with elevated MR were significantly more likely to have nodal and distant recurrence compared to those with low MR (*P*=0.0366 and *P*=0.0223, respectively); there was no difference in soft tissue recurrence (**Table 5**). The same comparison among the T2 group demonstrated a similarly notable increase in nodal recurrence (*P*=0.0366) but no difference in soft tissue or distant recurrence, although the difference in distant recurrence approached significance. Of note, patients who had recurrence in multiple locations were included in each respective group when assessing for differences in site of recurrence based on MR. There were no overall differences in recurrence in the T3 or T4 groups.

**Table 5:** Recurrence location among patients with T1 and T2 tumors separated by mitotic rate and ulceration.

			Recurrence location (% recurrence)		
MR	Ulceration	Recurrence (%)	Soft tissue	Lymph node	Distant
T1 (n=1580)					
<2	No	33 (2.4)	13 (39.39)	13 (39.39)	11 (33.33)
	Yes	4 (17.4)	2 (50.00)	2 (50.00)	1 (25.00)
≥2	No	13 (7.8)	4 (30.77)	4 (30.77)	5 (38.46)
	Yes	1 (5.9)	1 (100.00)	0 (0.00)	0 (0.000)
*P*	No	0.0007	0.1015	0.0366	0.0223
	Yes	0.3725	>0.9999	0.4987	>0.9999
T2 (n=644)					
<4	No	51 (11.7)	19 (37.25)	16 (31.37)	31 (60.78)
	Yes	10 (13.2)	4 (40.00)	4 (40.00)	6 (60.00)
≥4	No	25 (26.3)	8 (32.00)	10 (40.00)	13 (52.00)
	Yes	8 (22.2)	2 (25.00)	5 (62.50)	5 (62.50)
*P*	No	0.0004	0.1194	0.0366	0.0564
	Yes	0.3450	>0.9999	0.4987	0.3270

MR, mitotic rate

Given that it is the current recommendation that SLNB be offered to patients with T1b tumors (0.8-1 mm thick without ulceration or ≤1.0 mm with ulceration), we also characterized recurrence in patients with non-ulcerated T1 tumors with elevated MR who did not undergo SLNB (n=81, 49 of whom had thickness <0.8 mm) (13). Of the 81 patients, 7 (8.64%) experienced recurrence. Broken down further, 3.70% had soft tissue recurrence, 2.47% had nodal recurrence, and 2.47% had distant disease. Of note, 3 of 7 patients with recurrence had thickness <0.8 mm. In contrast, of the patients with non-ulcerated T1 tumors with low MR who did not undergo SLNB (n=1172), 26 (2.22%) experienced recurrence with 9 in the soft tissue (0.77%), 11 in the lymph nodes (0.94%), and 9 distant (0.77%).

**Figure 1:**
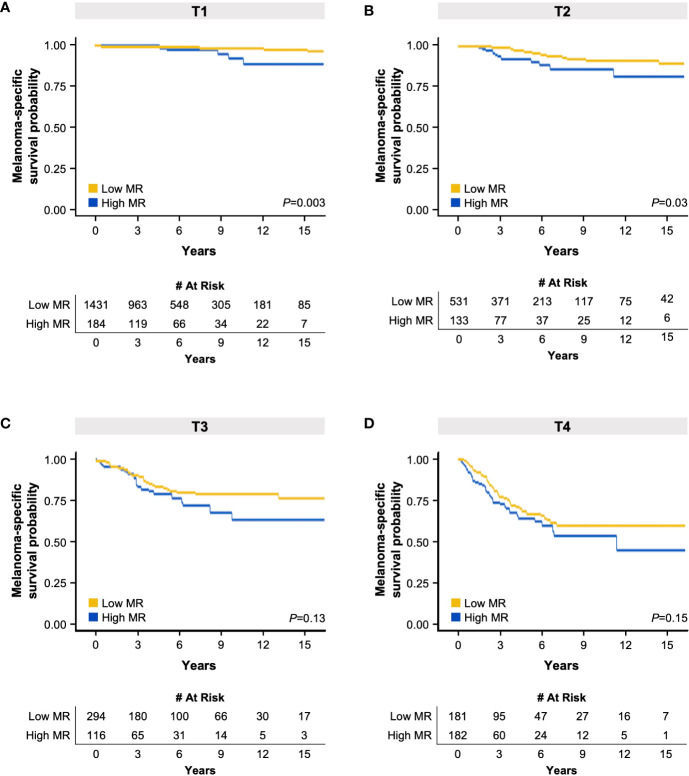
Kaplan-Meier melanoma-specific survival curves based on high or low mitotic rate (MR) in patients with T1 **(A)**, T2 **(B)**, T3 **(C)**, and T4 **(D)** melanoma. The tables below each respective graph indicate the number of patients at risk over the specified elapsed time (years) up to 15 years. .

With this knowledge, we subsequently assessed the impact of MR on survival within each T category using Kaplan-Meier analysis, similar to that performed by Kashani-Sabet et al. (11). Consistent with our previous findings, elevated MR was correlated with an adverse effect on survival compared to low MR in T1 and T2 tumors, but not in T3 or T4 tumors (**Figure 1**). Taken together, these analyses suggest that elevated MR, as defined by these pre-designated cut-points, is negatively correlated with survival in thin tumors, much like ulceration.

## Discussion

Multiple studies before and after AJCC8 have shown that increasing MR is negatively correlated with survival (4, 5, 9, 11). The removal of MR from AJCC8 was likely not because MR is not an independently predictor of melanoma outcomes, but because the previous inclusion of MR as a dichotomous variable in the AJCC7 staging system did not reflect its more dynamic role. In our study, we confirm that MR is a significant factor contributing to SLN positivity, recurrence, and, ultimately, MSM for patients with thin melanomas.

Research has suggested that MR has a dynamic, nonlinear impact on outcomes (7, 11). Multiple groups have attempted to identify the optimal means by which to group MR to best characterize its effect (3, 4, 9, 11, 18). In this study, we sought to validate the cut-points utilized by Kashani-Sabet et al. (≥2 mitoses/mm^2^ for T1, ≥4 mitoses/mm^2^ for T2, ≥6 mitoses/mm^2^ for T3, or ≥7 mitoses/mm^2^ for T4), as these authors provided strong evidence for these cut-points, which were obtained through multivariate analyses within training and validation models (11). To our knowledge, Kashani-Sabet et al. are also the only authors to specifically correlate MR with T category. A previous univariate analysis of stage I and II melanoma patients by Gershenwald et al. had previously demonstrated increased MR was significantly associated with increasing MSM by grouping MR into 0, 1, 2-3, 4-10, and ≥11 mitoses/mm^2^ independent of T category (9). Similarly, Tas and Ertuk determined that higher MR is a significant predictor of early relapse and unfavorable survival by assessing the effect of no/low (0-1 mitoses/mm^2^), medium (1.1-4.0 mitoses/mm^2^), high (5-9.9 mitoses/mm^2^), and very high (≥10 mitoses/mm^2^) MR (3). Rather than developing a potentially unique set of cut-points from our own database that may result in additional uncertainty about how to best utilize MR, we hoped that validation of the pre-determined cut-points derived from a large, seemingly strong study would contribute to efforts to reinstate MR as a prognostic factor in AJCC guidelines.

We found that elevated MR of ≥2 and ≥4 mitoses/mm^2^ was significantly associated with SLN positivity in T1 and T2 tumors, respectively. This is consistent with prior studies that have considered the cut-point of ≥2 mitoses/mm^2^ to be a critical marker (5, 19, 20). Piñero-Madrona et al., for instance, found that analysis of 141 patients by a single pathologist revealed increased sensitivity and specificity with 1.50 mitoses/mm^2^ and that ≥2 mitoses/mm^2^ was better correlated with overall and disease-free survival than 1 mitoses/mm^2^. Skochdopole et al. also found that elevated MR was a predictor of SLN positivity in T1 tumors through a query of the Surveillance, Epidemiology, and End Results database, although they noted a significant difference with MR ≥4 mitoses/mm^2^ rather than ≥2 mitoses/mm^2^ (4). Taken together, our study confirms that a cut-point of 2 mitoses/mm^2^ is important, specifically for thin melanomas.

In general, the assessment of the effect of MR on SLN positivity is limited by the fact that not all patients undergo SLNB, either due to AJCC guidelines at the time of diagnosis, patient choice, surgeon judgment, or a combination of these factors. Our study is beneficial in that it is encompasses a large number of patients who were treated at a single institution with a long history of melanoma care with relatively consistent care between providers.

SLNB is currently offered to patients with T1b tumors, which are 0.8-1.0 mm thick without ulceration or ≤1.0 mm thick with ulceration (13). Among our cohort, the percentage of positive SLNs among patients with non-ulcerated T1 melanomas with high MR (14.1%) is higher than the 8% incidence of SLN metastases in patients with lesions ≥0.8 mm cited by the ASCO-SSO guidelines (13). Our findings therefore suggest that SLNB should also be considered in patients with T1 tumors with an elevated MR even if they are <0.8 mm thick or without ulceration. The percentage of patients with T1 tumors who have an elevated MR is fortunately low (11.6% in our patient population), but these results suggest that surgical referral in this small group of patients can be important.

Our findings add to the literature because they not only validate and provide additional evidence for previous research demonstrating that 2 mitoses/mm^2^ is an important cut-point for thin melanomas, but also suggest that guidelines proposed by other studies require further refinement. For instance, there are a few key differences between our and Kashani-Sabet et al.’s conclusions. While Kashani-Sabet et al.’s multivariate Cox regression analysis suggested that MR may have a greater impact than ulceration, we found that MR had a significant but lower impact than ulceration (**Tables 2**, **3**) (11). Furthermore, our results did not demonstrate that elevated MR played a significant role in the outcomes of T3 and T4 tumors. This may be due to several reasons. First, it is possible that MR becomes less important with increasing tumor thickness. Second, perhaps the cut-points utilized for these thicker tumors were not optimal and different cut-points may result in notable differences. In addition, the insignificance may be due to the fact that our survival analyses are limited by the relatively low percentage of patients who had died from melanoma (7.0%, in contrast to 25.2% in the study by Tas and Ertuk) (3). Further research with greater cohort sizes will be necessary to best identify the optimal cut-points.

This study is largely limited by its retrospective nature, but this is somewhat counterbalanced by the fact that our data is collected in a prospective manner given our high volume of patients treated for melanoma at our institution. As a result, only 68 patients (2.2%) had to be excluded from analysis for incomplete data.

In conclusion, MR is an important prognostic factor for primary cutaneous melanoma and should therefore be considered for reincorporation into the staging system. Our findings demonstrate an association between elevated MR and MSM, as well as an increased risk of SLN positivity and recurrence in T1 tumors, independent of ulceration. SLNB should be considered for patients with tumors that have elevated MR ≥2 mitoses/mm^2^ even if they are <0.8 mm thick or without ulceration.

## Data availability statement

The original contributions presented in the study are included in the article/supplementary material. Further inquiries can be directed to the corresponding author.

## Ethics statement

The studies involving human participants were reviewed and approved by Yale Institutional Review Board. Written informed consent for participation was not required for this study in accordance with the national legislation and the institutional requirements.

## Author contributions

CLL, SA, KO, and JEC designed the study. HK and AG contributed to research discussion and refinement to the study plan. OB and WW performed the statistical analyses. CLL and OB analyzed the data. CLL drafted the manuscript. All authors contributed to the article and approved the submitted version.
